# Supplementary material for: Anxiolytic Treatment Impairs Helping Behavior in Rats
Source: Front Psychol. 2016 Jun 8;7:850. doi: 10.3389/fpsyg.2016.00850 (PMC4896909; doi:10.3389/fpsyg.2016.00850)
Supplement: Supplementary file 1 [file Data_Sheet_1.DOCX]

Supplementary material

1. Detailed methods for general linear model.
2. R code for general linear model
3. Detailed methods for reinforcement test
4. Matlab code for the null model testing for day-to-day reinforcement
5. Matlab code for power-matching bootstrapping analysis
6. The original latency data for all rats tested with a trapped rat in comma separated value format
7. The original latency data for all rats tested with a chocolate-containing restrainer in comma separated value format

Supplement A: Detailed Methods for general linear model

Opening latencies of each subject (16 per group) on each day (12 per subject) from each experimental group (5 trapped groups, 3 chocolate groups) were analyzed using a general linear model with the statistical software *R (R Foundation for Statistical Computing, Vienna, Austria, used under the General Public License)* and the R package "regress" (*David Clifford and Peter McCullagh, used under the General Public License).* The R code for the analysis (Supplement B) as well as the original latency data (Supplement F) are included below.

The general linear model contains the following terms for sources of variance: *treatment* (fixed effect, 5 nominal levels), *day* (fixed effect, 12 ordinal levels), *rat* (fixed effect, 16 subjects per group * 12 days * 5 groups = 960 nominal levels), and interaction between *treatment* and *day*. The final source of variance considered was *Vrat* (see next section), which accounts for correlations between latencies on different days within the same subject. Note that there were two missing data points (one from day 1 of one rat injected with low MDZ and one from day 2 of one rat injected with high MDZ), bringing the total number of analyzed values to 958.

*Rationale for and generation of Vrat.* The data are correlated due to the fact that many measurements come from the same subject. However, because of a learning effect over days (i.e. opening latency decreases over time but does not increase), the correlation between latencies on two different days within the same subject decays as the interval between the days increases. Therefore, individual between-pair correlation coefficients ($\alpha$) need to be generated for each interval in the data set. Vrat contains non-zero coefficients only for intervals within the same subject; for pairs containing opening latencies from two different subjects, there is no within-subject correlation and therefore the correlation coefficients are zero. Thus, *Vrat* is a 960*960 matrix with 12 non-zero values representing the correlation coefficients from the 12 possible day intervals that range from 0 to 11. The value of $\alpha$ for the day interval of 0 is 1.0 whereas $\alpha$ decays to 0.28 for the maximum interval of 11 days (see supplementary figure 1).

By generating *Vrat* with either an exponential decay or a linear decay and testing the log maximum likelihood estimate (LMLE) of the resulting models to compare their relative goodness of fit, we determined that an exponential decay is a better approximation. The $\alpha$ for each pair of opening latencies is therefore calculated as

$\alpha= e^{-\gamma\cdot d}$,

where $\gamma$ is a uniform correlation coefficient, constant across all pairs, that reflects the strength of correlation between latencies of two days within the same subject, and *d* is the absolute value of the difference between two days (e.g. latencies from day 1 and day 5 will have *d = 4*). We determined $\gamma$ for each experiment by comparing models of different $\gamma$ and selecting the value that resulted in the LMLE, as a larger LMLE indicates a better fit.

In a standard repeated-measures ANOVA, the correlation between days within the same subject is assumed to be constant across all intervals. However, since animals learn from their experiences, latencies on adjacent days are more correlated than are latencies separated by longer intervals. The matrix term *Vrat* offers the advantage of being able to accommodate smoothly changing correlation coefficients between pairs of days separated by increasing intervals. In sum, *Vrat* allowed us to account for experimental subjects’ changing their behavior through learning.

*Comparing hypotheses and testing significance of interactions.* The alternative hypothesis is stated as follows.

$latencies\sim(treatment+day+ rat +treatment*day + Vrat)$

The polynomial regression equation is the following:

$$Y_{i}=\beta_{1}*{treatment}_{i} + \beta_{2}*{day}_{i}+\beta_{3}*{treatment}_{i} *{day}_{i}+\beta_{4}*{rat}_{i}+\beta_{5}*\boldsymbol{Vrat}_{i,j}+\epsilon_{i} (i,j=1, 2, 3, \cdots, 960)$$

Here $\beta_{n}$ represents the respective regression coefficient calculated by regression analysis, $i$ refers to the opening latency out of 960 latencies, and $\epsilon_{i}$ represents the unobserved random error.

The null hypothesis, which states that there is no interaction between *treatment* and *day*, can be stated as follows.

$$latencies\sim(treatment + day + rat + \boldsymbol{Vrat})$$

The polynomial regression equation for the null hypothesis is therefore the following.

$$Y_{i}=\beta_{1}*{treatment}_{i} + \beta_{2}*{day}_{i}+\beta_{4}*{rat}_{i}+\beta_{5}*\boldsymbol{Vrat}_{i,j}+\epsilon_{i} (i,j=1, 2, 3, \cdots, 960)$$

We then calculated the statistical significance of the difference between the goodness of fit of the null and alternative hypotheses. To do this, we looked up the probability of the following term on a $\chi^{2}$ distribution with 4 degrees of freedom:

$$p=1-pchisq(2*({LMLE}_{alternative}-{LMLE}_{null}), df=4$$

The results are reported as the calculated χ^2^ value (see above), the degrees of freedom, and the corresponding probability.

The same analysis was performed on rats tested with a restrainer containing chocolate. However, since there were only three experimental groups (saline, high MDZ, low MDZ), the degree of freedom was two.

Differences in CORT levels were tested using MMA with ''day'' (1, 13) and “sampling point” (baseline, test, post) as the repeated measures. In addition, CORT *responses* (test value less baseline value) were analyzed with MMA with “day” as the repeated measure. Sidak tests were conducted for all post-hoc analysis. Pearson’s R was used to calculate the correlation between the number of door-openings and CORT responses on day 1 of testing. All statistical comparisons were conducted using SPSS (PASW 18).

Supplement B: R code for general linear model

Numbers in text are given for trapped rat condition. Numbers for chocolate condition are provided in the comments.

data <- read.csv("data.csv", header=TRUE) #read the data file

time <- c(data$D1, data$D2, data$D3, data$D4, data$D5, data$D6, data$D7, data$D8, data$D9, data$D10, data$D11, data$D12) #latencies from day1(D1) to day12(D12) are read

w <- !is.na(time); ndays <- 12; nrats <- 80 #two data points are missing, for chocolate groups, nrats=24

y <- time[w]

drug <- relevel(as.factor(rep(data[,1], ndays))[w], ref="uninjected") #treatments are read as factors (i.e. nominal)

rat <- as.factor(rep(data[,2], ndays))[w] #individual rat IDs are read as factors (i.e. nominal)

day <- rep(1:ndays, rep(nrats, ndays))[w] #days are read ordinally

d <- abs(outer(day, day, "-")) #the intervals between each pair of days are calculated

c(length(y), length(drug), length(rat), length(day), length(fday), dim(d)) #check the lengths of all the vectors

gammahat <- 0.116 #The uniform correlation coefficient. It is estimated by codes below under "Gamma evaluation" gammahat for chocolate groups is 0.13

Vrat <- outer(rat, rat, "==") * exp(-gammahat *d) #The matrix Vrat is produced with an exponential function

fit <- regress(y~day*drug, ~rat+Vrat, start=c(1,1,1), pos=c(1,1,1)) #data is fitted to the general linear model.

summary(fit) #result of fitting is displayed

ndlevs <- length(levels(drug)) #number of levels in treatment is read

K <- model.matrix(~day+drug)

fit0 <- regress(y~day+drug, ~rat+Vrat, start=c(1,0.1,1), pos=c(1,1,1)) # null model with no interaction

fit1 <- regress(y~day*drug, ~rat+Vrat, start=c(1,0.1,1), pos=c(1,1,1), kernel=K) # essentially the same as fit, but with kernel K

X2 <- 2*(fit1$llik - fit0$llik) #difference between the two models is plugged into a chi-square distribution

1 - pchisq(X2, df=ndlevs-1) #calculate p-value for interaction

###### Gamma evaluation

gamma <- seq(0.15, 0.08, -0.01) #gamma should be between 0 and 1. Previous testing showed that it should fall within 0.08-0.15.

llik <- matrix(0, length(gamma), 4) #the same model with different gamma values is reiterated and the likelihood of different models are compared

for(i in 1:length(gamma)){

Vrat <- outer(rat, rat, "==") * exp(-gamma[i]*d)

fit1 <- regress(y~fday*drug, ~rat+Vrat, start=c(1,1,1), pos=c(1,1,1))

llik[i,] <- c(fit1$llik, fit1$sigma)

}

plot(gamma, llik[,1], cex=0.5) #maximum likelihood of the model at different values of gamma are plotted, and the gamma value that results in the largest maximum likelihood is chosen

Supplement C. Detailed methods for reinforcement test

If a rat experiences opening the restrainer as rewarding, then door-opening behavior will be reinforced and the likelihood of opening on the *next day* will increase. Thus, reinforcement would be marked by a probability of opening on two sequential days (%SO) that is significantly higher than chance level. To test this, we compared the observed %SO to a distribution of chance %SO values generated with the following method.

To generate chance %SO values, a model was constructed that takes into account the effects of learning and also of individual differences. Rats may display non-associative learning over the course of testing sessions, putatively through processes that include habituation to the testing conditions, motor and perceptual learning, and conditioning. There are also individual differences between rats that might include motor, cognitive, and social differences. Together, these effects concentrate openings to later days in select rats, leading to high chance %SO levels. By incorporating the effects of learning and individual differences, the model produces realistic chance %SO values and allows a realistic estimate of the opening behavior on sequential days that would occur by chance, in the absence of reinforcement learning.

For each condition with N rats and 12 days, we created an observation matrix ***M*** with N rows and 12 columns. Opening observations from each condition were transformed into a binary distribution (1 for opening, 0 for not opening). The proportion of openings contributed by a rat (a measure of individual differences) was multiplied by the proportion of all openings that occurred on each day of testing among the group (a measure of learning). This product, after being normalized to the total number of openings in the group, is the probability that any given rat will open on any given day. Thus, we calculated an *N* by 12 matrix, ***P***, of probabilities for all instances of opening on day *j* by rat *i*. The matrix is given by:

$$\boldsymbol{P}_{i,j}=\frac{\sum_{k=1}^{N} \boldsymbol{M}_{k,j}\times\sum_{l=1}^{12} \boldsymbol{M}_{i,l}}{\sum_{k=1}^{N} \sum_{l=1}^{12} \boldsymbol{M}_{k,l}} (for i=1,2,\ldots,N;j=1,2,\ldots,12)$$

We then generated 10,000 binary matrices using the probabilities from ***P*** and calculated the %SO for each. From each distribution, we then estimated how extreme the observed %SO was compared to chance expectations by calculating a two-tailed p-value.

Supplement D. Matlab code for the null model testing for day-to-day reinforcement

ratnumber=16; %Define size of data matrices

iteration=10000; %Define number of iterations of null simulations

distpN11=zeros(1,iteration); %Create storage vector for probability of sequential opening from each simulation

indv=zeros(1,ratnumber); %Create storage vector for individual differences coefficients

daye=zeros(1,12); %Create storage vector for day-to-day differences coefficients

%Calculate differences coefficients for the data

for m=1:12

daye(m)=sum(data(:,m))/sum(sum(data));

end

for n=1:ratnumber

indv(n)=sum(data(n,:))/sum(sum(data));

end

%Iterations

for i=1:iteration

null=zeros(ratnumber,12); %Generate a null matrix that matches the data matrix

c11=0; c1deno=0; %Reset counters of sequential openings and openings

for m=1:12 %Generation of binary simulated observations

for n=1:ratnumber

np=rand;

if np>=daye(m)*indv(n)*sum(sum(data))

null(n,m)=0;

else null(n,m)=1;

end

end

end

for m=1:11 %Count sequential openings and total openings

for n=1:ratnumber

if null(n,m)==1

c1deno=c1deno+1;

if null(n,m+1)==1,

c11=c11+1;

end

end

end

end

pN11=c11/c1deno; %Calculate probability of sequential opening

distpN11(i)=pN11; %Store probability of sequential opening from this iteration

end

%%

cd11=0;c1ddeno=0; %Reset sequential opening and total opening counters

for m=1:11 %Calculate probability of sequential opening for the data

for n=1:ratnumber

if data(n,m)==1

c1ddeno=c1ddeno+1;

if data(n,m+1)==1,

cd11=cd11+1;

end

end

end

end

pD11=cd11/c1ddeno; %Calculate probability of sequential opening

%%

display(pD11)

display(mean(distpN11))

ExN11=0; %Reset extreme simulation counter

for i=1:iteration %Calculate number of simulations that are as extreme as the observation (two-tailed)

if abs(distpN11(i)-mean(distpN11))>=abs(pD11-mean(distpN11))

ExN11=ExN11+1;

end

end

pvalue11=ExN11/iteration; %Calculate p-value

display(pvalue11)

Supplement E: Matlab code for power-matching bootstrapping analysis

rawtestiteration=100; %number of bootstrapped samples

pvalue=zeros(1,rawtestiteration); %Storage for p%SOs from bootstrapped analysis

vec=1:1:16;

data=zeros(8,12); % Analogous as Supplement C

for a=1:rawtestiteration

index=datasample(vec,8);

for b=1:8

data(b,:)=raw(index(b),:);

end

ratnumber=8;

iteration=5000;

distpN11=zeros(1,iteration);

indv=zeros(1,ratnumber);

daye=zeros(1,12);

for i=1:iteration

null=zeros(ratnumber,12);

c11=0; c1deno=0; c0deno=0;

cd11=0;c1ddeno=0; c0ddeno=0;

for m=1:12 % Individual Differences

daye(m)=sum(data(:,m))/sum(sum(data));

for n=1:ratnumber

indv(n)=sum(data(n,:))/sum(sum(data));

np=rand;

if np>=daye(m)*indv(n)*sum(sum(data))

null(n,m)=0;

else null(n,m)=1;

end

end

end

for m=1:11

for n=1:ratnumber

if null(n,m)==1

c1deno=c1deno+1;

if null(n,m+1)==1,

c11=c11+1;

end

end

end

end

pN11=c11/c1deno;

distpN11(i)=pN11;

end

%%

for m=1:11

for n=1:ratnumber

if data(n,m)==1

c1ddeno=c1ddeno+1;

if data(n,m+1)==1,

cd11=cd11+1;

end

end

end

end

pD11=cd11/c1ddeno;

%%

ExN11=0;

for i=1:iteration

if abs(distpN11(i)-mean(distpN11))>=abs(pD11-mean(distpN11))

ExN11=ExN11+1;

end

end

pvalue(a)=ExN11/iteration;

end

Supplement F. The original latency data for all rats tested with a trapped rat in comma separated value format

drug,rat,D1,D2,D3,D4,D5,D6,D7,D8,D9,D10,D11,D12

saline,R1,40,40,40,40,40,40,40,40,40,40,40,40

saline,R2,40,40,25,12,2,0,0,1,0,0,0,0

saline,R3,19,40,40,40,40,0,4,0,0,0,0,2

saline,R4,10,40,40,40,40,40,40,40,40,40,40,40

saline,R5,6,40,40,40,40,40,40,40,4,40,40,3

saline,R6,14,36,38,2,4,0,0,0,0,0,0,0

saline,R7,40,40,40,40,40,40,40,40,4,40,40,40

saline,R8,40,40,40,40,40,40,35,40,0,4,4,3

saline,R9,40,40,40,40,40,40,6,11,7,2,0,1

saline,R10,40,40,40,6,40,5,10,1,0,2,4,2

saline,R11,40,40,40,40,40,40,40,40,40,40,40,40

saline,R12,40,40,40,40,40,40,40,40,40,40,40,28

saline,R13,40,40,40,5,11,1,1,1,0,0,0,0

saline,R14,40,40,40,40,40,40,40,14,40,40,40,40

saline,R15,40,40,40,40,40,40,40,40,40,40,40,40

saline,R16,40,40,40,40,40,40,40,40,40,40,40,40

uninjected,R17,40,40,40,40,40,40,40,40,40,40,40,40

uninjected,R18,40,40,40,40,6,0,4,0,2,2,0,0

uninjected,R19,40,40,40,40,40,40,40,40,40,40,40,40

uninjected,R20,40,40,40,40,15,40,40,4,40,2,40,6

uninjected,R21,15,12,3,1,1,0,0,1,1,1,0,1

uninjected,R22,40,40,40,40,40,7,5,2,1,0,2,3

uninjected,R23,40,40,15,5,5,0,1,0,0,0,0,1

uninjected,R24,40,40,40,40,40,40,40,40,40,40,40,40

uninjected,R25,40,40,7,25,33,2,2,0,0,0,0,0

uninjected,R26,16,40,40,36,17,0,1,35,2,1,0,0

uninjected,R27,40,40,40,40,4,13,3,3,40,5,33,1

uninjected,R28,40,40,8,1,8,4,0,1,0,2,3,2

uninjected,R29,40,40,40,40,40,40,40,40,40,40,40,40

uninjected,R30,40,40,40,40,40,40,40,40,40,40,40,40

uninjected,R31,20,4,2,16,4,1,3,0,0,0,1,0

uninjected,R32,40,40,40,40,40,40,40,40,40,40,40,40

lowMDZ,R33,40,40,39,18,40,1,1,1,0,1,2,4

lowMDZ,R34,40,40,40,40,40,4,40,40,40,40,40,40

lowMDZ,R35,3,40,40,40,40,40,40,40,29,40,40,7

lowMDZ,R36,3,22,40,6,40,40,40,40,40,40,40,40

lowMDZ,R37,7,5,40,40,40,40,40,40,40,40,40,40

lowMDZ,R38,16,40,40,40,40,40,40,34,2,2,1,0

lowMDZ,R39,40,40,40,40,40,40,40,40,40,40,40,40

lowMDZ,R40,,40,40,40,40,40,40,40,40,40,40,40

lowMDZ,R41,40,40,40,40,40,40,40,40,40,40,40,40

lowMDZ,R42,5,4,40,40,40,40,40,40,40,18,26,1

lowMDZ,R43,40,40,39,3,3,1,1,13,0,0,0,0

lowMDZ,R44,40,14,28,40,40,40,40,40,9,0,1,0

lowMDZ,R45,40,40,40,40,40,40,40,40,40,40,40,40

lowMDZ,R46,40,2,2,0,0,1,2,0,0,1,0,0

lowMDZ,R47,40,40,40,40,40,9,40,40,40,40,40,5

lowMDZ,R48,40,5,40,35,40,40,35,40,40,40,32,40

highMDZ,R49,40,40,40,40,40,40,40,40,40,40,40,40

highMDZ,R50,40,,40,40,40,40,40,40,40,40,40,40

highMDZ,R51,40,6,40,40,40,40,40,40,40,40,40,40

highMDZ,R52,40,40,40,40,40,40,40,40,40,40,40,40

highMDZ,R53,40,40,40,40,40,40,40,40,40,40,40,40

highMDZ,R54,40,40,40,40,40,40,6,40,40,40,40,24

highMDZ,R55,40,40,40,40,40,40,40,40,40,40,40,40

highMDZ,R56,40,19,15,40,1,40,40,23,5,40,2,21

highMDZ,R57,40,40,40,40,40,40,40,40,40,40,40,40

highMDZ,R58,40,40,40,40,40,40,40,40,40,40,40,40

highMDZ,R59,40,4,40,40,40,40,40,40,40,2,40,40

highMDZ,R60,40,40,7,40,40,40,40,40,40,40,40,40

highMDZ,R61,40,40,40,40,40,40,40,40,40,40,40,40

highMDZ,R62,40,40,40,29,40,40,40,40,40,0,32,40

highMDZ,R63,40,40,40,40,40,40,40,40,40,40,40,40

highMDZ,R64,40,40,40,40,40,40,40,40,40,40,40,40

nadalol,R81,40,40,40,40,40,40,40,30,15,5,6,2

nadalol,R82,40,40,40,40,40,40,40,8,1,3,3,0

nadalol,R83,20,40,18,8,40,40,40,40,40,37,40,40

nadalol,R84,40,40,40,40,40,40,40,10,3,0,0,1

nadalol,R85,40,40,40,40,40,9,4,5,3,1,1,0

nadalol,R86,40,40,40,40,30,40,7,10,1,0,1,4

nadalol,R87,11,12,40,40,40,40,40,40,40,40,40,40

nadalol,R88,40,40,40,40,2,40,40,40,40,40,40,40

nadalol,R89,40,40,4,40,40,40,40,40,40,40,40,40

nadalol,R90,40,40,40,40,40,40,15,40,40,40,11,0

nadalol,R91,40,40,40,40,40,40,40,40,40,40,40,40

nadalol,R92,6,40,6,12,40,40,40,23,11,0,3,1

nadalol,R93,40,40,40,7,9,4,0,0,0,1,1,2

nadalol,R94,11,40,40,21,1,14,2,4,0,0,0,1

nadalol,R95,40,40,40,40,40,40,40,40,40,40,40,40

nadalol,R96,40,40,40,40,40,40,1,40,40,40,40,40

Supplement G. The original latency data for all rats tested with a chocolate-containing restrainer in comma separated value format

drug,rat,D1,D2,D3,D4,D5,D6,D7,D8,D9,D10,D11,D12
LowMDZ,R1,40,18,10,1,1,1,0,1,5,3,0,0
LowMDZ,R2,40,40,40,40,40,40,40,40,40,40,40,16
LowMDZ,R3,40,40,40,6,6,3,0,1,5,0,1,0
LowMDZ,R4,40,29,40,40,5,12,0,0,0,0,0,0
LowMDZ,R5,40,40,40,40,30,12,5,1,0,1,1,40
LowMDZ,R6,40,40,40,40,40,40,40,40,40,40,40,40
LowMDZ,R7,40,8,40,36,11,6,1,1,0,1,4,0
LowMDZ,R8,40,40,40,31,2,0,1,1,0,1,1,2
HighMDZ,R9,40,40,40,40,40,40,40,40,40,40,40,40
HighMDZ,R10,40,40,40,40,38,2,1,1,1,0,1,0
HighMDZ,R11,40,40,40,40,40,40,40,40,40,40,40,40
HighMDZ,R12,40,40,40,40,40,40,40,40,40,29,2,1
HighMDZ,R13,40,40,40,0,40,40,40,40,40,40,40,40
HighMDZ,R14,40,2,2,40,0,2,1,1,0,0,0,0
HighMDZ,R15,40,40,5,42,3,2,2,1,2,1,2,1
HighMDZ,R16,40,40,40,20,40,13,7,2,0,1,1,1
Saline,R17,5,40,40,40,40,40,40,40,40,40,40,40
Saline,R18,4,40,40,40,40,40,40,40,40,40,40,40
Saline,R19,40,40,40,40,40,40,40,40,40,40,40,40
Saline,R20,40,40,40,40,40,40,40,40,40,40,40,40
Saline,R21,40,40,40,40,40,40,40,40,40,40,40,40
Saline,R22,40,40,40,40,40,40,40,40,40,40,40,40
Saline,R23,40,40,40,12,40,40,40,40,40,10,40,40
Saline,R24,15,26,40,20,5,5,2,2,0,1,0,0
